# Can an Open-Label Placebo Be as Effective as a Deceptive Placebo? Methodological Considerations of a Study Protocol

**DOI:** 10.3390/medicines7010003

**Published:** 2020-01-02

**Authors:** Leo Druart, SaraEve Graham Longsworth, Carole Rolland, Maïa Dolgopoloff, Hugo Terrisse, Jean-Luc Bosson, Nicolas Pinsault

**Affiliations:** 1 Physiotherapy Department, University Grenoble Alpes, 38000 Grenoble, France; 2 Techniques pour l’Évaluation et la Modélisation des Actions de Santé (ThEMAS), Techniques de l’Ingénierie Médicale et de la Complexité (TIMC), Unité Mixte de Recherche (UMR), Centre National de la Recherche Scientifique (CNRS) 5525, Université Grenoble-Alpes, 38000 Grenoble, France

**Keywords:** open label placebo, pain, cold pressor test, ethics, clinical trial, placebo

## Abstract

**Background:** Placebo has been studied for many years and is ever-present in healthcare. In clinical practice, its use is limited by ethical issues raised by the deception entailed by its administration. **Objective:** To investigate whether, when given detailed information about pain and underlying placebo mechanisms, subjects will have a response similar to that of those subjected to a procedure in which they receive a conventional placebo treatment. **Methods:** The study is designed as a non-inferiority randomized, parallel with a nested crossover trial. In addition, 126 subjects without any known pathology will be included. They will be randomized into two groups. Each subject will undergo three Cold Pressor Tests (CPT): calibration, condition of interest (deceptive placebo or educated placebo), and control. Our main judgment criterion will be the comparison in pain intensity experienced on the visual analog scale between the two CPTs with placebo conditions. **Results:** This study will allow us to rule on the non-inferiority of an “educated” placebo compared to a deceptive placebo in the context of an acute painful stimulation. It is another step towards the understanding of open-label placebo and its use in clinical practice. **Conclusions:** This study has been approved by the ethics committee in France (2017-A01643-50) and registered on ClinicalTrials.gov (NCT03934138).

## 1. Introduction

The placebo effect has been studied for decades and is present in medical and paramedical care. Though placebo treatments have shown their efficacy in numerous pathologies, their use in common practice is limited because of ethical issues. Placebos are utilized mostly as a methodological tool in control groups to demonstrate the efficiency of studied therapies.

Placebo was initially described as an inert substance or therapeutic procedure devoid of any pharmacological effect. Benedetti highlights an important nuance and describes the placebo not only as the inert substance but “its administration within a set of sensory and social stimuli that tell the patient that a beneficial treatment is being given” [1]. Indeed, the placebo effect can be in part defined as a “psychobiological phenomenon occurring in the patient’s brain after the administration of an inert substance, or of a sham physical treatment along with verbal suggestions (or any other cue) of clinical benefit” [2]. Although there is no consensus on the taxonomy of the terms, the placebo effect and the placebo response are not interchangeable terms—the latter being the sum of all non-specific effects taking place after administration of a placebo treatment and the placebo effect being precisely the psychosocial and psychobiological non-specific effects [1,3,4,5]. However, some authors suggest different definitions [6].

Many factors have been identified as potentially influencing the placebo effect and several mechanisms have been studied that elicit it [1,4]. The two main mechanisms implicated are learning and expectations. Other mechanisms such as social learning, memory, concentration, reward, and decreased anxiety [7] have also been studied and can either be classified into learning processes or expectations’ modulation. A third mechanism of keen interest in recent research explores the genetics involved in the placebo effect [8]. Several genes have been identified in the dopaminergic, opioidergic, endocannabinoidergic, and serotoninergic pathways as modulators of a subject’s placebo response.

Throughout recent decades, endogenous placebo mechanisms have been investigated. They refer to endogenous cascades triggered by expectancy, learning, and their combination, whereas exogenous mechanisms depend on pharmacologically induced effects [6]. Levine brought up this hypothesis for the first time in 1978 and since then many studies have confirmed it [9,10]. The authors described an endogenous response triggered by a positive expectancy [11]. Studies using fMRI [12,13] and PEI [14] have shown common mechanisms for placebo-induced and opioid-induced analgesia, particularly the activation of common cerebral regions. These findings have been confirmed and further studied as shown by recent reviews [15,16]. There is now solid evidence identifying the areas of the brain in play during placebo analgesia for example.

The expectation modulation mechanism includes the patient’s expectations and that of the practitioner. A group of subjects hoping for a positive effect of a placebo treatment is more likely to experience the expected improvement of the symptoms [17]. The trial proposed in the present article attempted to modulate the subject’s positive expectations using their understanding of underlying placebo mechanisms.

### 1.1. The Use of Placebo in Contemporary Clinical Practice

Numerous studies have explored the use of placebo treatments in clinical practice in industrialized countries. In the United Kingdom, in 2013, a survey involving interviews with 783 physicians [18] showed that 78% used placebo treatments at least once a week. In addition, they indicated that at least 97% of physicians had used them, most often impure ones (meaning derived from its original use yet inert on the symptom treated [5]), at least once in their careers. Pure placebos such as saline injection were very rarely utilized. In Denmark, the 2003 study by Hrobjartsson and Norup showed that 86% of general practitioners reported using at least one “intervention with no specific effect on the condition to be treated but a possible non-specific effect” in the previous year [19]. The main reason given was to avoid confrontation with the patient. In Tilburt et al.’s. study in 2008 [20], 334 interns and 345 rheumatologists in the United States were interviewed. Results demonstrated that 55% of practitioners used a placebo at least once during the previous year and 46% at least two or three times a month. Again, the majority of prescriptions were for impure placebos. Of these, 70% were antibiotics, which raises issues for the patient and for the community as well. More recently, a review from Linde and collaborators [21] confirmed these results on a large-scale meta-analysis. Firstly, we can see large intervals of responses (from 29 to 97% used placebo treatments at least once in their career) that seem to indicate that the concepts are quite differently defined in each study. Secondly, we can see that placebo treatments are widely used in practice and that these treatments are more often impure than pure.

### 1.2. Ethical Concerns

The ethical issues raised by the deception associated with placebo treatments limit its use. Indeed, “most physicians agree that the placebo effect plays a significant role, but that the use of placebo is often associated with uncertainty regarding the ethical dimensions of whether and how to communicate the use of a placebo to the patient” [22]. In addition, in the field of research, opinions are divided and, in most countries, there are no official regulations.

A first option is the use of placebo as a lure. That is, the inert nature of the given treatment is hidden from the patient in order to provide a placebo effect. Some authors such as Foddy justify lying to or omitting information from the patient because the treatment is administered in the patient’s interest [23]. However, this represents a hindrance to the patient’s autonomy and strengthens the doctor’s paternalistic relationship with the patient. In this context, the patients cannot give informed consent nor can they refuse the treatment.

Moreover, this deceptive use of placebos can harm the therapist–patient relationship when the deception is discovered or revealed. “When a patient finds that a real illness was treated by a fake drug, the doctor–patient relationship will rupture, and may have long-term consequences on the patient’s capacity to trust any medical advice” [24]. However, placebos can be a useful solution when there is no effective treatment available.

Considering previous arguments, in November 2006, the American Medical Association adopted an ethical policy prohibiting the use of placebo associated with deception in clinical practice. Added to that, many authors favor the use of open-label placebo [25] mostly because of the ease in manipulating patients that is underlined by the discovery of physiological mechanisms involved in the therapeutic ritual [4].

### 1.3. Placebos without Deception

However, if the patient takes a placebo treatment with full disclosure in regard to the nature of the treatment, will the therapeutic effect observed in the previously cited studies still be present? This question had started to raise interest as early as 2003. Authors such as Aulas and Rosner conducted a clinical trial on non-blind placebos as treatments for anxiety, in subjects suffering from depression, to answer this question [26]. This trial was a good starting point yet suffered from important methodological limitations. To overcome these limitations, Kaptchuk et al. designed one of the first randomized controlled trials on placebos without deception. This study compared an open-label placebo (OLP) to a no treatment arm with patients suffering from irritable bowel syndrome (IBS) [27]. In the OLP group, patients knew “that the placebo pill was an inactive (i.e., «inert») substance like a sugar pill that contained no medication.” About 15 min were set aside to provide information about placebo. The symptom improvement in this group was clinically and statistically superior to the control condition. Results were surprisingly high: “finally, the percentage of patients reporting adequate relief (59%) is comparable with the responder rates in clinical trials of drugs currently used in IBS,” demonstrating that, in a context of persuasive reasoning, OLP may show effectiveness comparable to the established treatments in IBS.

Other studies have confirmed Kaptchuk’s team’s hypothesis [28]: placebos without deception seem to improve a range of clinical symptoms while maintaining patient autonomy and trust in the physician. Recent reviews found OLP treatments to be clinically effective compared to no-treatment groups [29] on several conditions including: IBS, depression, allergic rhinitis, chronic low back pain, and attention deficit hyperactivity disorder (ADHD).

Nonetheless, it is still unclear how OLPs work [30] and how to increase their effectiveness. One variable that is to be considered is the rationale given during its administration.

Such explanations are starting to be studied more and more. For example, Locher et al. [31] compared OLP and deceptive placebos with a focus on the rationale given to the patients. The research hypothesis was that the educated placebo would still be less effective than the deceptive placebo. Other studies such as Schaefer et al.’s RCT [32] looked at information as a trigger of positive expectancy in allergic rhinitis.

This protocol’s hypothesis is that knowledge and understanding of the mechanisms at work in the placebo effect will increase the intensity of the open placebo response towards a non-inferiority compared with deceptive placebo. This is the reason the group with the variable of interest is called “educated” placebo. This study will be the first, to our knowledge, to test for non-inferiority between an educated placebo and a deceptive one.

## 2. Materials and Methods

### 2.1. Objectives

The main objective of this study is to investigate whether or not an educated placebo is non-inferior to a deceptive placebo treatment on treating acute pain intensity. Secondary objectives of the study include verifying the superiority of both placebo treatments with a no-treatment Cold Pressor Test (CPT). We will also assess the effect of both placebo treatments on anxiety. This study also aims to measure whether or not the educational video was effective in transmitting knowledge about the placebo effect.

### 2.2. Design

We will conduct a non-inferiority randomized, parallel with a nested crossover trial comparing educated open-label placebo to deceptive placebo. This monocentric study will be carried out by two licensed physiotherapists in a University Hospital near the campus.

Written informed consent will be obtained from subjects prior to their participation in the study. The investigation will consist of a single two-hour session. Participants will receive a 20€ participation compensation.

**Figure 1:**
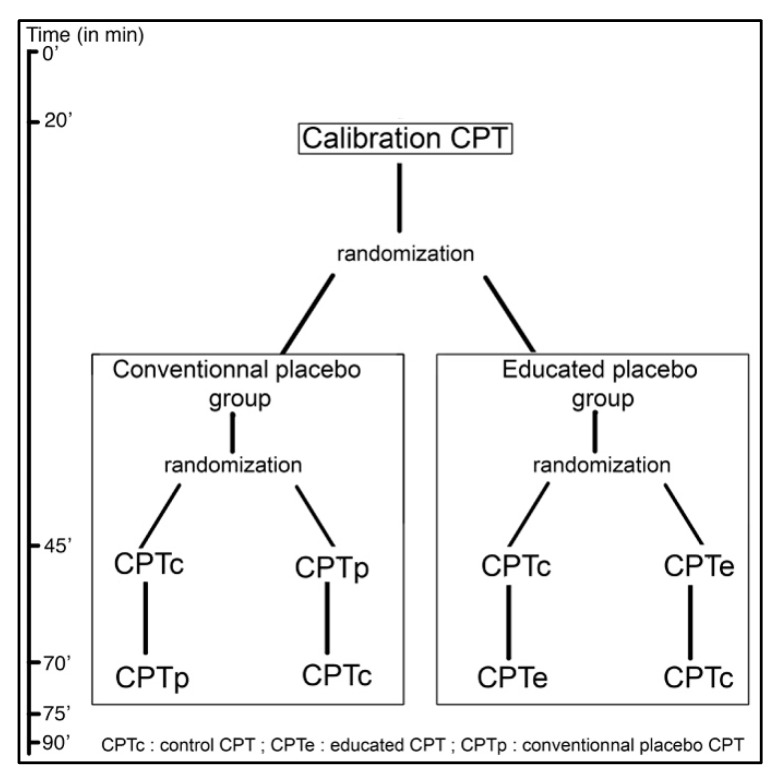
CPT order diagram.

The subjects meeting the inclusion criteria (detailed below) will be randomized into two groups: (1) educated placebo or (2) conventional/deceptive placebo. Each will be subjected first to a calibration CPT, and then, in a randomized order, will receive the CPT under the condition of interest (either the Conventional placebo CPT (CPTp) or the Educated placebo CPT (CPTe)) and a Control CPT (CPTc). This is represented in Figure 1.

Regarding blinding, investigators will not be blind as they deliver the treatment openly in the educated group. Patients in the deceptive placebo group will be blinded. However, patients in the educated placebo group will not be due to the nature of OLP. The analysis will be done blindly as the analyst will not know which group received which treatment.

### 2.3. Assessment

The primary end point will be the difference in pain intensity, assessed on a 100-point visual analogue scale (VAS), between the educated OLP and the deceptive placebo treatments at the end of the placebo condition CPT. We have chosen pain intensity as opposed to pain threshold because it involves less motivational and cognitive components [33].

The main secondary end point will be, for each subject, the difference in pain intensity, also assessed by VAS, between open or deceptive placebo versus no-treatment control. Secondarily, a questionnaire (inspired by Hughes et al.’s data [34]) measuring knowledge regarding the placebo effect will be completed by all the subjects after the study and also before the educational video for the educated placebo group. In addition, all the subjects will complete another questionnaire about their perception of the investigators and of the study [35]. Blood pressure (BP) and heart rate (HR) values will be obtained before, during, and after each CPT by means of an automatic blood pressure monitor. Finally, subject’s anxiety will be evaluated through heart rate variability. Before the CPT treatment, an expectancy questionnaire will be used [36].

We expect to find a non-inferiority between both groups on the main end point. Considering the nature of the dependent variables and the choice of the painful stimulation, we expect a high interindividual variability. This has been limited as best as possible by the cross-over conducted in both arms of the study.

### 2.4. Subjects

Participants are to be recruited via advertisements for a study of a painkiller cream, on the Grenoble University campus. Inclusion criteria are: being aged between 18 and 40 years old, registered to the national healthcare, having understood and signed the written consent. Non-inclusion criteria were the following: legal impossibility to participate to the protocol (i.e., pregnant women, people deprived of their liberty) or affections modifying the painful stimulus used (any known pathology affecting the venous, arterial, or lymphatic system, diabetes, cardiac affections, asthma, frostbite on the hand, epilepsy, hand arthritis, lupus erythematosus, and allergic reactions to the cream, under pain regulation medication).

All medication that could impact pain sensation, such as painkillers or psychotropics, have to be stopped three weeks prior to experiment, as will alcohol in the 24 h prior to the experiment.

Socio-demographic information will be collected in order to control the impact of these variables in the representation of the placebo effect and consequently expectations towards the treatments.

### 2.5. Ethical Considerations Regarding the Study Protocol

During conceptualization of the protocol, several ethical questions were raised. The first was about the information given while administering the cream as a deceptive placebo. While it was first considered to present it as “an analgesic cream”, this was deemed too much of a deception and unethical. The committee in charge of ethics agreed on the use of the sentence “This is a cream useful for treating pain” as it was argued that was true due to its non-specific effect.

Secondly, the choice of reaching 7/10 as a threshold on the VAS was debated. It was argued that this was a high level of pain. However, the duration of this intense pain was deemed short enough to present little risks and be a probable situation in a clinical setting. The treatments used in the case of pain rated over 7/10 are also those with the most side-effects and thus these types of intense pain experiences would benefit most from OLP treatments. The time frame required to obtain a score of 7 on the VAS with a CPT is still within a reasonable duration.

Lastly, among the often-used pain stimulation methods, the choice of using a CPT was also questioned. This point is discussed in the discussion below.

### 2.6. Material

The Cold Pressor Test (CPT), Dip Cooler RU 200 from Techne^®^ (Cole Parmer, Staffordshire, United-Kingdom) will deliver the pain stimulus. The duration of the immersion will be calibrated during the first CPT and maintained for an identical time in the two others. More on the use of the CPT is presented below.

Tablets will be used to fill out the assessments as well as watch the videos. The content of the educational video will be detailed below. The control video (about the history of hygiene and Semmelweis) was taken from a well-known video broadcasting website with written consent from the creator.

The placebo treatment used for both groups is a neutral cream with no active pharmacological substance: CremaFluid Phytomedica^®^ (Laboratoires Phytomedica, Aix-en-Provence France). It will be presented as a placebo cream in group 1 (educated placebo) and as a painkiller in group 2 (deceptive placebo).

### 2.7. Randomization

Randomization was carried out in several steps. Firstly, an analyst from inside the team wrote the code to get a randomized spreadsheet of groups and CPT orders. The rest of the procedure was conducted by the research team’s staff not involved in the protocol. Another analyst was asked to attribute to each group an unidentifiable name in order to blind the analysis of results (group A and group B) as well as choose a random seed. Lastly, another person made individual, numbered, and sealed envelopes with the randomization results inside. This entire process was done with no involvement from the investigators. The envelopes are kept under lock and key.

This process allows for preservation of the blinding of the analyst (not knowing which treatment group A and B received) and maintains the investigator in the dark regarding group attribution until the envelope is opened.

### 2.8. CPT Procedure

Each volunteer will be initially subjected to a calibration test according to the CPT procedure and then to the experimental tests. After this, a debriefing and two questionnaires will conclude the inclusion.

Each CPT procedure is as follows. Every CPT is preceded by a 3 min monitored resting period where heart rate and respiratory rate are measured. Blood pressure is measured at the end of this 3 min baseline assessment. The hand and forearm temperature will be checked on both sides for reference. The non-dominant hand and distal third of the forearm will be immerged into the CPT at 1 °C +/− 1 °C. The forearm length submerged in the cold-water tank will be determined as follows: 1/3 of the distance between the ulnar styloid and the tip of the olecranon. The elbow will be placed on the outer rim of the water tank and the hand relaxed with no contact with the bottom of the tank.

For the calibration CPT, patients will be asked to record the intensity of their pain on the VAS every 5 s approximately. When the intensity exceeds 7/10 on the VAS, the subject will be told to remove his or her hand from the water. The time at which this event occurs will be referenced as (*t*). For both of the other CPTs, patients will be asked to keep the hand in the tank during (*t*) seconds and complete a VAS as soon as the hand is taken out of the water.

In between each CPT, a 20 min pause is mandatory and skin temperature is checked before the next CPT. If needed, a tank of warm water at approximately 25 °C can be used to warm up the hand until it reaches contralateral temperature.

### 2.9. Part I: Calibration

The protocol can be described in three different phases described below. The first phase is the calibration. Subjects are greeted by the main investigator who will explain in detail the nature of the pain stimulation applied and inform subjects of their rights regarding data privacy and protocol interruption. Inclusion and non-inclusion criteria are checked before signing a written consent. Once consent is obtained, the randomization envelope is opened to allocate subjects to a group and determine the order of the CPTs. The calibration CPT can then start following the procedure stated above in part 2.8.

This calibration will enable us to use a constant duration for the immersion of the hand in the water for the two remaining CPTs, and will allow us to ensure that the intensity of pain is homogeneous.

### 2.10. Part II: Experimental CPTs

During the break between calibration and the second CPT, the subject will receive the information related to the group they are randomized into. For the deceptive placebo group, the video will describe the history of hygiene and correct method for washing hands. For the educated placebo group, the video will be an educational video about placebo, its physiology, and the use of OLP. Movies are similar in terms of duration (~11 min), image quality, and format. After both videos, there will be a time where questions can be answered by the investigators. The educated placebo group will fill out a questionnaire regarding their knowledge of the placebo effect before watching the educational video.

The test phase will be divided into two parts in a random sequence to compensate for habituation effect: deceptive placebo CPT (CPTp) or educated placebo (CPTe) and control CPT (CPTc). For the CPTp and CPTe, two milliliters of the neutral placebo cream are applied beforehand following a standardized procedure: four round-trips on the forearm along its submerged part and on the dorsal and palmar side of the hand and an expectancy questionnaire is filled out. The immersion time (t) in cold water will be that previously calibrated.

While applying the neutral cream, the examiner will explain to the deceptive placebo group, that “it is a cream effective in fighting cold-related pain.” In the Educated placebo condition, the examiner states that it is “a placebo cream, containing no active substance that is effective in fighting cold-related pain. All the mechanisms shown in the previous film will be at work. In a way, your brain will be secreting the active components itself.”

Under the control condition, no cream will be applied.

The examiner will be aware that the same placebo cream is used for the two groups, in order to simulate real situations in which the practitioner administering or prescribing the placebo does so with full knowledge of its presence. The patient will not be blinded in the OLP group and will be blinded in the deceptive placebo group. The analyst will be blinded to group allocation.

### 2.11. Part III: Subject Debriefing

After all three CPTs, all subjects are asked to fill out the questionnaire on their knowledge of the placebo effect. One group (deceptive placebo group) is naive to this questionnaire and the other is filling it out again after having viewed an educational video approximately 40 min ago. This will allow for controlling information retention. Subjects then fill out a questionnaire on their perception of the research hypothesis as well as their perception of the investigators.

After the inclusion is finished, a debriefing session is conducted by the main investigator in order to explain the research protocol and the deception if one has taken place. This is done in order to be sure patients understand what the research was about and to answer any questions left by the protocol. This debriefing also allows to be sure the pain stimulation was well lived through.

**Figure 2:**
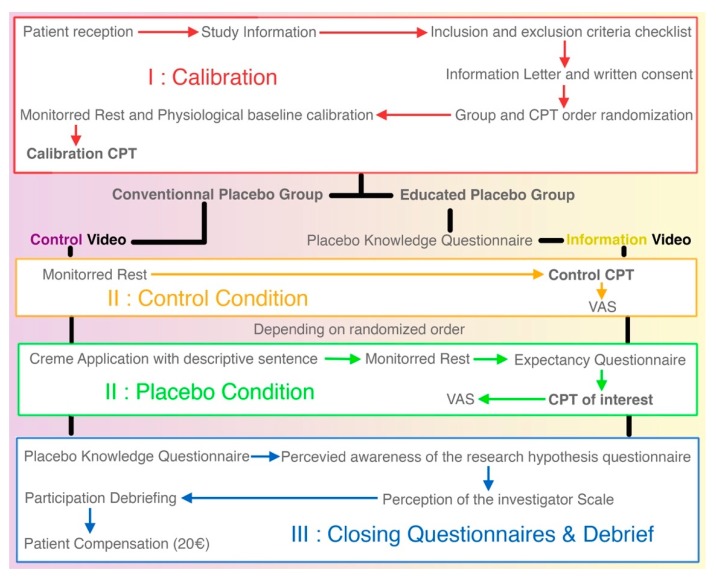
Detailed study flow for a subject. From top to bottom for an animated version of this figure, see: http://bit.ly/Placethic-Figure.

Figure 2 is a detailed flow chart of the research protocol. From top to bottom are the three phases of the protocol. The white boxes are the procedures common to both groups.

### 2.12. Sample Size

As previously stated by the FDA and confirmed by the MCID recommended by Myles et al. [37], we set the margin of non-inferiority at 10 mm. Based on a 21.9 mm standard-deviation of the VAS score^38^ and assuming an alpha of 0.05, the minimum sample size required to achieve a power of 80% to reject the inferiority null hypothesis was 60 per group. Adding to that a 5% margin of estimated non-usable data the total number of subjects will be of 126.

Streff et al. [38] had a similar utilization of experimental pain in their study using a CPT on healthy subjects and a visual analog scale (VAS) to estimate pain. In the dataset they present, a standard deviation of 21.9 mm is calculated.

### 2.13. Statistical Analysis

We will perform the statistical analysis with alpha = 0.05. Unless stated otherwise, all tests will be bilateral. We will perform the tests with an Intention To Treat population: all participants will be analyzed in the group in which they were initially randomized.

Missing data on the primary endpoint will be imputed by multiple imputation if between 5% and 20% of the measurements are missing. If less than 5% are missing, we will not impute the missing data. If more than 20% of the data are missing, the results will be interpreted with caution. We will not perform missing data imputation on the other endpoints.

We will test the non-inferiority of the Educated Placebo compared to the Conventional Placebo using the unilateral 95% confidence interval of the difference of the pain intensity. The non-inferiority margin is set to 10 mm on the VAS. We will estimate the confidence interval by linear regression. The linear regression will account for the pain intensity without placebo and the sequence of the CPTs. As an Intention to Treat analysis can be biased when evaluating a non-inferiority [39], we will also test the non-inferiority with a per protocol analysis: subjects will be included in the group only if there were no deviations from the protocol. If the results of the two analysis are discordant, we will interpret the results with caution.

We will also test the superiority of the Conventional Placebo compared to no-treatment by a cross-over ANOVA. Similarly, we will test the superiority of the Educated Placebo compared to no-treatment by a cross-over ANOVA. We will test the other between group differences by Student’s *t*-test for two independent samples.

We will test the effect of the education in the Educated Placebo group by Student’s *t*-test for paired samples comparing the knowledge about Placebo before and after the educational video.

This study will allow us to rule on the non-inferiority of an educated placebo compared to conventional placebo in the context of an acute painful stimulation. We have highlighted the need to study the different determinants increasing the placebo effect, and in particular the understanding of underlying placebo mechanisms. This study is the first step in a series of research works that could allow an ethical use of placebo in clinical practice.

Analysis will be conducted on Stata software, version 15 (StataCorp LLC, College Station, TX, USA) or higher.

## 3. Results

This research protocol has been approved by the Ethical Committee (Comité de Protection des Personnes Sud Ouest et Outre mer III) on the 28th of February 2018 under the identification 2017-A01643-50 and registered on ClinicalTrials.gov under the identification number NCT03934138. Funding has been acquired through the APICIL Foundation to conduct the investigation and compensate patients. Recruitment has started during May 2019 and will take place until the required number of subjects is recruited.

Pre-testing was conducted in order to assess logistical needs and train the investigators. Slight modifications were adopted such as the reduction of intervals between each VAS during the calibration CPT from 10 to 5 s. The instructions for the CPT were also made more precise to include instructions in regard to not touching the bottom of the tank or moving the hand during the procedure. During these pre-tests, we were able to better estimate the time needed for set-up during which the cooling of the CPT is time-consuming. The average time for inclusion is closer to one hour and a half compared to the initial two hours announced to the subjects. However, this extra time allows for a technical margin.

**Figure 3:**
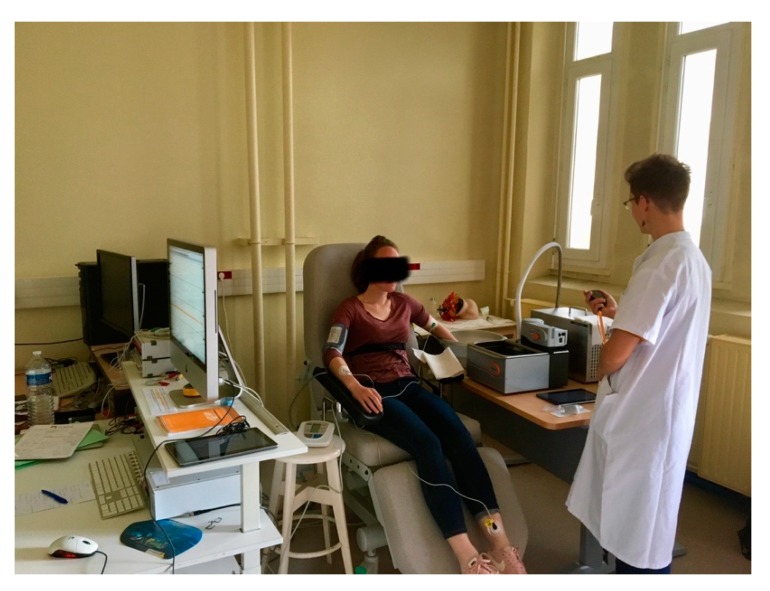
Clinical trial set-up during a CPT test.

The final set-up is illustrated in Figure 3. Monitoring was conducted through the computer to the left. Patients had to their right the CPT tank as well as the warm water if needed. Investigators were wearing lab coats over casual clothing.

## 4. Discussion

### 4.1. Methodological Justifications

Many of the methodological choices made during conceptualization of the protocol need to be argued and explained. The first choice made during conception was to conduct a non-inferiority trial. Such trials are useful in asserting the non-inferiority between two procedures when one has a clear advantage over the other whether it being economical, iatrogenic, or ethical. In the present protocol, the educated placebo has a distinct ethical advantage over the deceptive placebo, and this justifies the choice of resorting to a non-inferiority analysis.

At the time of obtaining ethical authorizations in 2016, too few open-label placebo studies were published and none were conducted in France to justify its use on patients in the eyes of ethical committees. Therefore, the ethical committee authorized a trial conducted on healthy volunteers with an experimental pain. This is another step towards justifying OLP use with patients.

Experimental pain modalities are numerous although common techniques involve either heat, cold, or electrical stimulations. In this case, we opted for the use of a CPT with water at 1 °C. This method is broadly used and has well described modalities towards its use as a pain stimuli [38,40,41,42]. Although CPTs present a high inter-subject variability, the cross-over design will mitigate any effect this would have on the results of the secondary endpoint variables. Setting the temperature at 1 °C allows for average times of 40 s before reaching pain tolerance [43]. Regarding setting the VAS limit at 70/100 before taking the hand out of the tank, this is justified because of the patients we wish to treat in future protocols. These patients suffer from intensive pain (>7/10) that requires special treatments often associated with strong adverse events. These patients would benefit the most from innovative solutions to treat pain such as OLP.

Another choice made for this protocol is the use of VAS as the main evaluation criterion. This measure of pain intensity is not influenced by the cognitive and motivational components that influence pain tolerance or pain threshold or duration of immersion [33]. Its ease of use and of comprehension is another advantage [44]. The numerous secondary criteria will allow to assess the anxiety subjects felt via measure of their heart and respiratory rate. Expectancy and perception of the study and the investigator will be measured with questionnaires. Both groups will fill out a placebo knowledge questionnaire with no prior explanation on the effect. Only the educated placebo group will take this test again once the protocol is finished. This will allow us to control the understanding of the educational video.

The choice of using a cross-over methodology when comparing placebo to no-treatment allows us to control for each subject and each group with a no-treatment measure. This is a methodological strength of the protocol. The randomization of the order in which these control and placebo CPTs are done allows for adjusting on habituation that other studies have found [45]. Each CPT takes place after a 20 min wash-out period where vasodilatation may return to its baseline state. This is verified by a check that hand temperature is comparable on both hands as well as having the VAS return to 0 before performing a new test.

One crucial element of the protocol resides in the choice of using a video capsule to deliver the rationale as well as constructing the rationale. To do so, we contacted authors in several other open-label placebo trials to use their scripts as a starting point. Once this was done, we took the most common misconceptions found by Hughes et al. [34] and discussed them in the video. To give credibility to the video and reinforce the positive expectations of patients watching the video, we described several landmark articles and trials about placebos and open label placebos as well as gave feedback from patients from those trials. The video was illustrated with several everyday life examples. A cartoonist was hired to give visual graphical illustrations over which a text was read. To ensure proper understanding, any questions patients could have regarding the video are answered. The control group is also presented with a video to watch. This video is presented as a video to distract subjects from the painful experience before starting the interventional phase of the study. Both videos are similar in regard to length and format.

During this trial, the subject’s knowledge about the placebo effect needed to be measured in order to check for effectiveness of the education. To our knowledge, there are no questionnaires specifically validated for our population to this end. However, a recent study [34] constructed a 15-item questionnaire to survey placebo knowledge among trial participants. We adapted this questionnaire to use in our study (available in French and English in the Supplementary Files S1 and S2). After coming up with a first version of the questionnaire, we pre-tested it with 10 subjects meeting our inclusion criterion and five people older than the age limit set in the protocol. Pre-tests were conducted via cognitive interviews. The questions were clear for all subjects and allowed us to proceed with this questionnaire in our trial. During the trial, the need for a naive response from the educated placebo groups imposes to fill the questionnaire before the CPT of interest. This may be considered as part of the intervention as this condition is not present in the deceptive placebo group (to complete Figure 2 we added in the Supplementary Files a table comparing the interventions received in both groups). In any case, every element of the questionnaire is addressed in the video and analysis will check for any influence of the questionnaire on the placebo response.

### 4.2. Future OLP Research

As stated during the 2019 SIPS conference, future research on open-label placebo will be faced with several challenges in order to justify a potential use in a clinical setting. Among these challenges is the investigation of the mechanisms behind open-label placebos’ efficacy and what triggers exist that modulate this efficacy. Current hypotheses revolve around the patient–therapist relationship, the treatment in itself and/or the rationale given to the patients. This study will allow for gaining insight regarding the latter. The modalities of the educational intervention, explained and argued in the previous subsection, will allow for interesting discussion once the results are obtained.

Another challenge future research needs to take into account is to conduct investigations on subjects that are not healthy subjects. This protocol helps towards justifying the use of OLP with patients once used with healthy subjects with no risks and shows potential benefits. At the time of conceptualization of this protocol, too few studies allowed for ethical justification of the use of OLP with patients.

One of the major difficulties in regard to OLP research is the difficulty in using control groups as a reference. Past studies have used no treatment or treatment as usual groups as controls. This study suggests an original methodology with two parallel groups as well as an intra-group control. This allows for intra-subject control as well as comparison between a no treatment condition and a treatment condition. Of course, this means that the statistical analysis must show, in order to conclude a non-inferiority with clinical relevance, that both the deceptive placebo and the open-label placebo are statistically superior to the no treatment condition while also finding the open-label placebo condition to be non-inferior to the deceptive placebo. This methodological challenge of having a valid control group seems to justify the necessity in publishing protocols allowing for a detailed peer-review process. This allows going in-depth into the process used to elicit a placebo response as well as give a detailed account of the context present during the inclusions.

Lastly, there persists a difficulty in the blinding of the different people involved. To be close to a clinical setting, it is impossible to blind the patient and the therapist. However, an independent assessor could help in blinding the assessment. This was not done in this protocol for material reasons. Instead, the primary outcome is self-reported and the analysis is done blindly by the statistical team.

To address these multiple challenges and avoid discrediting future OLP research, we argue that there is a need to give in-depth explanations of what happens during research protocols that elicit placebo responses whether they are open or not. However, the current scientific publication process often restrains authors towards the number of words used in an article. This is done for obvious reasons, yet does not allow for the exhaustive detail to be given in regard to the protocols and their justifications. This is why it seems crucial to encourage authors to publish their detailed research protocols and methodological considerations either in separate articles or in formats with a less restrained approach to the word limits.

In regard to this protocol, depending on the results, future research will be conducted to investigate the cognitive levers associated with the rationale that can increase OLP effectiveness. Comparisons between an optimized open-label placebo and several treatments in patients affected with persistent pain such as fibromyalgia or low back pain will also be designed and carried out.

## Supplementary Materials

The following are available online at https://www.mdpi.com/2305-6320/7/1/3/s1, File S1: Questionnaire Placebo French Version, File S2: Questionnaire Placebo English Version.
