# Supplementary material for: Can an Open-Label Placebo Be as Effective as a Deceptive Placebo? Methodological Considerations of a Study Protocol
Source: Medicines (Basel). 2020 Jan 2;7(1):3. doi: 10.3390/medicines7010003 (PMC7168289; doi:10.3390/medicines7010003)
Supplement: Supplementary file 1 [file medicines-07-00003-s001.zip › medicines-600924 supplementary proof done/Materials - Questionnaire Placebo English Version.pdf]

Self-assessment about placebo effects:

Instructions: Check the correct answers. There can be zero to all answers correct. The questionnaire consists of 17 questions spread over 3 pages.

Do you know about the placebo effect?

- Not at all
- I have a couple notions
- I have already heard about it
- Yes, I know what it is

1. **The placebo :**
   - Only has an effect on people who believe in it
   - Only works if the patient ignores that the treatment is a placebo
   - Has an effect if the patient knows it’s a placebo

2. **The mechanism of the placebo effect is**
   - Non-existent
   - Psychological
   - Psychological et physiological

3. **Placebo treatments are only effective on patients who are lying about their symptoms**
   - True
   - False

4. **A paracetamol tablet used to treat pain is a placebo**
   - True
   - False

5. **The placebo effect works in part through patient’s expectancy**
   - True
   - False

6. **The placebo effect is effective only with optimistic patients**
   - True
   - False

7. **Placebo treatments can help in treating pain**
   - True
   - False

8. Placebo treatments can’t resolve symptoms requiring specific medications

- o True
- o False

9. Analgesic placebo treatments only work on “imaginary” pain (i.e. pain that is not caused by a lesion or disease)

- o True
- o False

10. A tablet with no pharmacological substance is a placebo

- o True
- o False

11. The placebo effect work thanks to the context in which the care is administered

- o True
- o False

12. Physiological changes, such as the secretion of chemical molecules, occur in the brain when you receive a placebo

- o True
- o False

13. The placebo effect are imaginary and have effects only on our psyche and not on our body

- o True
- o False

14. The placebo effect only occurs during experiments, in clinical research

- o True
- o False

15. A placebo tablet can have secondary effects

- o True
- o False

16. There are no placebo effects during conventional medical treatment

- o True
- o False

17. The colour, shape and packaging of a placebo tablet can affect its effectiveness

- o True
- o False
