# Supplementary material for: Can an Open-Label Placebo Be as Effective as a Deceptive Placebo? Methodological Considerations of a Study Protocol
Source: Medicines (Basel). 2020 Jan 2;7(1):3. doi: 10.3390/medicines7010003 (PMC7168289; doi:10.3390/medicines7010003)
Supplement: Supplementary file 1 [file medicines-07-00003-s001.zip › medicines-600924 supplementary proof done/Materials - Questionnaire Placebo French Version.pdf]

Auto-évaluation à propos des effets placebos :

<u>Consignes :</u> Cochez les réponses correctes. Il peut y avoir d'aucune à et toutes les réponses justes. Le questionnaire comporte 17 questions réparties sur 3 pages.

Connaissez-vous le placebo ?

- Non pas du tout
- J'ai quelques notions
- J'en ai déjà entendu parler
- Oui je sais ce qu'est le placebo

1. **Le placebo :**
   - A un effet seulement chez les personnes qui y croient
   - Fonctionne uniquement si le patient ignore qu'il s'agit d'un placebo
   - A un effet si le patient sait qu'il s'agit d'un placebo

2. **Le mécanisme de l'effet placebo est**
   - Inexistant
   - Psychologique
   - Psychologique et physiologique

3. **Les traitements placebo ne sont efficaces que sur les patients qui mentent à propos de leurs symptômes**
   - Vrai
   - Faux

4. **Un comprimé de paracétamol utilisé contre la douleur est un placebo**
   - Vrai
   - Faux

5. **Les effets placebo fonctionnent notamment grâce aux attentes des patients**
   - Vrai
   - Faux

6. **Les effets placebos ne sont efficaces que chez les personnes optimistes**
   - Vrai
   - Faux

7. **Les traitements placebo peuvent traiter des douleurs**
   - Vrai
   - Faux

8. **Les traitements placebo ne peuvent pas résoudre des symptômes nécessitant une médication précise**
   - Vrai
   - Faux

9. **Les traitements placebo dits « antalgiques » ne soulagent que les douleurs imaginaires (i.e. douleurs n'étant pas liées à une lésion/maladie)**
   - Vrai
   - Faux

10. **Un comprimé sans substance pharmacologique est un placebo**
    - Vrai
    - Faux

11. **Les effets placebo fonctionnent grâce au contexte dans lequel est administré le soin**
    - Vrai
    - Faux

12. **Des modifications physiologiques, comme la sécrétion de molécules chimiques, ont lieu dans le cerveau quand vous recevez un placebo**
    - Vrai
    - Faux

13. **Les effets placebo sont imaginaires et n'ont d'effets que sur notre psychique et non sur notre corps**
    - Vrai
    - Faux

14. **Les effets placebo n'ont lieu que lors des expériences, en recherche clinique**
    - Vrai
    - Faux

15. **Un comprimé placebo peut avoir des effets secondaires**
    - Vrai
    - Faux

16. **Il n'existe pas d'effets placebo lors d'un traitement médical classique**
    - Vrai
    - Faux

17. La couleur, la forme et le conditionnement d’un comprimé placebo peuvent modifier son efficacité

- Vrai
- Faux
